# Delusion of Jinn Possession in a Young Man With Schizophrenia: Case Report and Impact of Including a Religious Leader in Patient Care

**DOI:** 10.1155/crps/4021081

**Published:** 2026-05-08

**Authors:** Aamna Asif, Nathan H. Hwang, Rabia Asif, Fatima Asif, Ahmad El Gamal, Robert T. Rubin

**Affiliations:** ^1^ Internal Medicine Residency, Los Robles Regional Medical Center, Thousand Oaks, California, USA; ^2^ Psychiatry Residency, Community Memorial Healthcare, Ventura, California, USA; ^3^ Western University College of Osteopathic Medicine, Pomona, California, USA; ^4^ St. George’s University School of Medicine, West Indies, St. George, Grenada, sgu.edu; ^5^ MS Program, Shari’ah Department, Faculty of Islamic and Arabic Sciences, Al-Azhar University, Cairo, Egypt, azhar.edu.eg; ^6^ Department of Psychiatry and Biobehavioral Sciences, David Geffen School of Medicine at UCLA, Los Angeles, California, USA, ucla.edu

**Keywords:** imam, Islam, jinn, Muslim, religious delusions, ruqyah, schizophrenia

## Abstract

Spiritual beliefs of many ethnic and religious groups can play a major role in the maintenance of physical and mental health. Integration of spiritual understanding into medical treatment can enhance patient outcomes in many specialties, especially psychiatry. As the Muslim population grows worldwide, the integration of spiritually and culturally sensitive mental healthcare with Islamic principles becomes increasingly important for optimum treatment outcomes. Herein, we discuss the inclusion of spiritual care in the treatment of a young Muslim man with schizophrenia who presented with the belief of being possessed by a jinn, a supernatural being capable of possessing or harming humans. In the context of a biopsychosocial‐spiritual model of care, we focus on how Islamic principles augmented our patient’s treatment through involvement of an Islamic religious leader (imam) to expertly address this patient’s religious‐based beliefs. Continuing to integrate spirituality broadly into mental health care should enrich our understanding of human nature and the patient experience and result in better outcomes through holistic medicine.

## 1. Introduction

We present a case of a young Muslim man diagnosed with schizophrenia who had the belief of being possessed by a jinn, a supernatural being capable of possessing or harming humans. Our concern was whether standard psychiatric treatment would suffice, without formally addressing his religious beliefs through an Islamic spiritual leader. During the course of treatment, an imam was added to the clinical care team, with beneficial results.

Whether a religious belief that may be considered an overvalued idea, including being possessed by a spirit, is a matter of religious faith and perhaps part of a cultural norm, or is a delusion symptomatic of psychosis, has been at times difficult to discern. Pierre [1] suggested that certain characteristics of an overvalued religious belief other than content; for example, the conviction, preoccupation, and impact on functioning of the individual with the belief, as well as its cultural influence, may be the salient aspects of differentiating a faith‐based belief from a psychotic delusion. The perspective of the clinician also is important; for example, Irmak [2] opined that hallucinations in schizophrenia may be false interpretations of real sensory images formed by demons existing in a demonic world. Other clinicians may dismiss the concept of demons and demonic possession out of hand.

Demons exist in all major religions as supernatural beings capable of possessing or harming human beings; in Islam they are referred to as jinn. The Qur’an describes jinn as living in an unseen world (al‐ghayb) parallel to our own. Unlike humans, who are made of dried clay, God created jinn from the fire of a scorching wind [3]. Jinn exist in a parallel dimension to our world, with the main difference being that while jinn are able to move between the two dimensions, humans cannot [4]. Jinn share some characteristics with humans, including free will and the ability to think. According to the Qur’an, their free will allows them to choose obedience to God or to become misguided disbelievers, much like humans [5]. A subtype of jinn is the shaytaan (English cognate, satan), misguided disbelievers tasked with leading humans to deviate from the righteous path and the word of God [6, p 113]. It is believed that each person is assigned a jinn that can influence them to deviate from God’s path (for a further brief but comprehensive review of jinn in Islam, refer to [4].)

Spiritual beliefs, including the role of jinn, can significantly shape how some Muslims perceive and respond to mental health challenges [7]. In some cultures, the belief persists that jinn possession or influence can cause mental health symptoms similar to those occurring in depression, anxiety, psychosis, and schizophrenia [8]. Imams and other spiritual healers can play a crucial role in distinguishing true mental health disorders from jinn possession and potentially providing spiritual treatment through practices like ruqyah.

Ruqyah broadly translates as a method of healing and protection through the recitation of the Qur’an [9]. During ruqyah, an imam recites specific Qur’anic verses to remove jinn from the afflicted individual. Ibn Taymiyyah [10] a 13th‐century Islamic scholar, extensively wrote on the spiritual causes of mental health symptoms, including the harmful effects of *jinn* possession. While he emphasized treatment through Qur’anic recitations, he also advocated for medical interventions [10]. Contemporary Islamic teachings encourage the use of medical approaches, as noted in the hadith:

“*Make use of medical treatment, for Allah [God] has not made a disease without appointing a remedy for it, with the exception of old age*”[11].

Contemporary Islamic scholars promote a comprehensive approach to mental health, integrating both spiritual and medical treatments.

For Muslims, practices like reciting the Qur’an can alleviate anxiety and depression and support overall well‐being [12]; for example, an Islamic supplication states, “O Allah, make the Qur’an the spring of my heart, the light of my chest, the banisher of my sadness and the reliever of my distress” [13]. Mental health professionals who understand these religious beliefs can incorporate them into treatment plans, ensuring spiritually sensitive, evidence‐based care. Imams play an essential role by promoting mental health awareness through sermons, advocating for patients in healthcare settings, and assisting in culturally relevant decision‐making [7].

## 2. Case Presentation

The Ventura County Medical Center Institutional Review Board does not require submission of case reports for approval. The following case has been de‐identified as much as possible, and all HIPAA and other confidentiality requirements have been adhered to. CARE guidelines were followed in the writing of the report.

A male patient in his mid‐20s was admitted to the inpatient psychiatric unit for the sixth time, owing to suicidal ideation. He reported perceiving auditory commands from a jinn to harm himself in various ways, including stabbing himself with a knife, hitting himself in the head with stones, tying a rope around his neck, and engaging in physical altercations with police with the intent of being shot and killed. He also reported a burning pain throughout his body, originating from the jinn’s fiery form.

The patient’s mother indicated that her son was raised in an Arabic‐speaking, Middle Eastern country and practiced Islam. At age 13 he was sent to the United States by his emotionally and physically abusive father. The patient’s mother reported that her son had been sexually assaulted by an adult male acquaintance while living in his country of origin. On arrival in the United States, he knew only Arabic and was a practicing Muslim. At age 15 he began experiencing auditory hallucinations and exhibited social withdrawal, anhedonia, and avolition. He stated there was a jinn inside him that would not allow him to pray, read the Qur’an, or complete his activities of daily living. As well, the jinn often commanded him to kill himself. Further information from the family was not available.

While hospitalized, the patient expressed fear that if he were to be discharged, the jinn would cause him to commit suicide. During interviews, he would switch from speaking from his point of view to speaking as the jinn. When asked how he became possessed by the jinn, the patient (as the jinn) stated, “He (the patient) did something shameful with his genitalia and that’s how I possessed him.”

On mental status examination, the patient was of average height, with unkempt facial hair. He was alert, calm, cooperative, and oriented to time, place, and his situation. His speech was slow, with constricted tone and mild latency. His affect was markedly flat. He had no suicidal or homicidal ideation of his own. His thought process was largely goal‐oriented and organized, but he was consistent in the belief he was possessed by a jinn, which instructed him to harm himself and others. He did not elaborate on the shameful thing the jinn claimed he had done with his genitalia. He had impaired judgment and insight regarding his psychiatric condition.

Physical examination was within normal limits. An MRI of the brain did not show any abnormalities. Blood count and blood chemistry values also were unremarkable, other than mild deviations in white blood cell count. The patient tested negative for potential sources of organic psychosis, with normal thyroid hormone, hepatitis B and C antibody, nontreponemal antibody, and C‐reactive protein values.

During his hospitalization, the patient often wore a t‐shirt and sandals and neglected his grooming unless reminded to attend to his hygiene. He ambulated slowly, dragging his feet. He would greet hospital staff in the hallways and was calm and cooperative with psychiatric interviews. He continued to indicate to staff that the jinn instructed him to harm himself and others—the jinn would whisper these commands to him throughout the day, and during interviews, he was noted on occasion to respond to internal stimuli.

The patient indicated his family had taken him to an Islamic leader to have ruqyah performed to rid him of the jinn, but it was not effective because “the imam didn’t do it for long enough.” The patient’s mother confirmed that the ruqyah occurred, but the imam did not think a jinn was causing the patient’s symptoms at that time. As an outpatient, he previously had been prescribed clozapine, but it was discontinued because of intermittent adherence, persistence of psychotic symptoms, and risk of myocarditis.

While in the hospital, he was treated initially with olanzapine, lithium (for occasional, intense suicidality), propranolol, and gabapentin, along with psychotherapy as tolerated, frequent interactions with nursing staff, and inpatient group activities. Despite medication adherence, he continued to voice concerns to hospital staff and other patients that the jinn was instructing him to harm himself. He additionally was prescribed haloperidol and quetiapine, following which his avolition and anhedonia improved, but he continued to report auditory hallucinations of the jinn’s commanding him to carry out acts of violence toward himself and others.

After the patient had spent 25 days in hospital with limited improvement, the psychiatric team believed it would be beneficial to involve a religious leader. Within the broad concept of biopsychosocial‐spiritual care [14], and with the patient’s approval, an imam (co‐author Ahmad El Gamal) with experience in ruqyah was contacted to assess the patient. Given that the imam was not local, a Zoom meeting was arranged, during which the imam asked the patient a few questions, including why he believed there was a jinn inside him. At one point, the patient switched to speaking as the jinn, stating he was 700 years old, but without a change in the tone of his voice (a sign of jinn possession—see Section 3).

After a 20 min conversation with the patient, the imam told both the patient and the psychiatric team there was no sign of a jinn, and he read verses from the Qur’an to the patient. The patient initially stood up, paced the room, and stated the Qur’an recitation was burning the jinn (a common phenomenon during ruqyah when a jinn is believed to be present). The imam, utilizing knowledge specific to Islamic belief and based on their conversation and the patient’s actions, educated the patient on why there was no evidence of a jinn residing within him. The imam used specific examples from the interview and the patient’s actions to support his claim.

As the imam continued, the patient gradually relaxed and became calm and attentive for the remainder of the 30 min session. After the session, the patient stated he felt comfort while hearing the Qur’an and found benefit from the session. In subsequent interviews with the psychiatric team, he indicated fewer concerns about the jinn. He still believed he was possessed but stated, “The *jinn* lets me pray now.” His final diagnosis was schizophrenia, and he was discharged on haloperidol, quetiapine, and lithium to a short‐term crisis rehabilitation facility in stable condition, with plans for transfer to a continuing care facility.

The following year the patient was again readmitted, owing to continued auditory hallucinations instructing him to end his life. He had attempted suicide in various ways, including hitting his head against a lamp, cutting his wrists, trying to run into traffic, and trying to take control of a police officer’s firearm to shoot himself. He stated he was hearing the voice of the jinn again, instructing him to harm himself and others. At one point, the jinn had instructed him to attack his mother and burn down their home. The jinn intermittently attempted to choke him, making medications more difficult to swallow. The severity of the jinn’s commands had worsened over the 3 weeks prior to admission, despite the patient’s purported adherence to his medication regimen.

During this hospitalization, he again was treated at various times with haloperidol, aripiprazole, clozapine, lithium, lamotrigine, fluoxetine, mirtazapine, gabapentin, and clonazepam. Intramuscular medications were administered as needed for agitation. He prayed each day, which appeared to help him gain partial control over his thoughts of harming himself and others. As well, he found comfort in interacting with Arabic‐speaking nursing staff. After about 2 months his symptoms began to stabilize, and he was discharged to a continuing care facility with only residual, nondistressing auditory hallucinations.

## 3. Discussion

We performed a selective review of the medical literature on psychiatric patients claiming jinn possession to evaluate the effectiveness of involving Muslim religious leaders in their care. Table 1 presents reported cases of claimed *jinn* possession in which both a religious leader and mental health professionals were involved in their care. Of the nine cases, five were women; five were between ages 15 and 30; and eight were from Asian, Middle Eastern, or East African countries with deep roots in Islam. Notable is the range of psychiatric/neurological diagnoses, with ruqyah often being performed and having mostly positive results. In the first case, the imam decided there was no jinn possession, and the patient was given straightforward psychiatric treatment; in other cases, ruqyah had a strong positive effect; and in case 7 the patient had a brain infection, which was not affected by ruqyah.

**Table 1:** Nine cases of claimed jinn possession involving both a religious leader and psychiatric services.

Reference numbers	Age years	Sex	Country of origin	Presenting symptoms	Nature of jinn belief	Ruqyah done?	Jinn possession confirmed?	Elements considered in jinn possession	Psychiatric/medical diagnoses	Psychiatric/medical treatments	Treatment results	Comments
[15]	49	M	Somalia	Neglect, apathy, disinhibited behavior, low mood, delusions, thought insertion	Patient and family believed he was possessed by jinn	No	No	Imam ruled out jinn possession and indicated patient had mental health problems	Schizophrenia	Antipsychotic medication	Patient discontinued medication	Patient readmitted to psychiatric unit
[16]	25	F	Pakistan	Anger, agitation, anxiety, poor sleep, auditory and visual hallucinations of jinn, panic attacks, delusions, episodes of confusion	“I am possessed by jinn, I don’t need any medication.” Family agreed with jinn possession	No	No	Unknown	Delirious mania	Quetiapine and clonazepam	Patient did not follow up	History of bipolar disorder in sibling Similar episode 1 year earlier; patient saw an aamil (spiritual healer) and was given “a special arm band” and “holy water to drink.” Effectiveness not stated Patient refused further medical care No collaboration between clinicians and spiritual healer
[17]	15	F	South Asia	Suicide attempt after auditory hallucination of jinn telling her to jump off stairs	Patient and family believed patient was possessed by jinn	?	No	Unknown	Questionable psychosis	Patient in an arranged marriage Psychologist addressed jinn directly, jinn said he was angry at patient’s violent treatment by in‐laws, and suicide attempt was their punishment Psychotic symptoms abated	Patient did not return for medical treatment Family took her to traditional healer who accused her of faking the jinn; family then abused her even further	Healthcare team believed patient’s claim of being possessed by a jinn was not a psychiatric symptom, but a culturally acceptable way of expressing personal suffering—a veiled protest against abuse by her in‐laws
[18]	25	F	Iraq	Social withdrawal, uncommunicative, anorexia	Family believed patient was possessed	Yes	Unknown	Unknown	Severe depressive illness	Electroconvulsive therapy (ECT)	ECT did not improve symptoms	Symptoms improved after ruqya. Patient recalled not being able to initiate behaviors while “possessed”
[19]	(wife age 50)	M	Pakistan	Social withdrawal, poor sleep, agitation, night terrors, violent toward wife	Allah revealed to wife in a dream that her husband was possessed	Yes	Yes	Patient became agitated and had jerking movements after reading Qur’an	Depression	Paroxetine	Slight improvement in mood	After several readings of the Qur’an during ruqya, the jinn left the patient, and his mental state improved
[9]	22	M	Pakistan	Severe left eye pain, elevated heart rate	Not stated	Yes, multiple	Inconclusive	Too few symptoms (tiredness, headaches)	Cluster headache, migraine	Morphine	Only temporary pain relief with morphine; headaches worsened	After each ruqyah, headache pain subsided Weekly ruqyah sessions alleviated patient’s need for morphine
[18]	35	F	Not stated	Fever, confusion, incomprehensible speech, jaundice, epileptic seizures	Patient and family believed she was possessed	Yes	Yes	Unknown	Cerebral malaria	Pharmacotherapy for cerebral malaria	Patient recovered	Patient initially diagnosed with typhoid fever; because of possession belief, she did not finish antibiotic treatment Her condition deteriorated after ruqya; later diagnosis of cerebral malaria was treated successfully
[20]	60	M	Of Asian origin	Psychomotor retardation, altered mental status, agitation, visual hallucinations, paranoid delusions, anxiety, insomnia	Patient believed Jinn entered his home and were moving around him Family believed patient was possessed by evil spirit	Yes, twice	No	Unknown	Not specified	Unspecified hypnotic for insomnia	Significant improvement in mental state	Antipsychotic use deferred; patient instead given sessions with traditional healers Patient terrified by first session, but prayers from the second healer helped him Discharged from hospital 2 weeks later with no follow‐up
[21]	21	F	Sri Lanka	Episodic emotional lability, “possession” states several times a day	Patient and family believed she was possessed by demons	Yes	Not specified	Unknown	Not specified	Not specified	Patient recovered	Patient recovered after dropping out from psychiatric services and seeking help through religious rituals

In summary, Table 1 indicates heterogeneous collaborations between religious leaders and psychiatric clinicians in the contemporary care of patients claiming jinn possession. The results of these cases, along with our case presentation, emphasize the importance of considering the close collaboration of a religious leader in the mental health care of patients with strong Islamic beliefs, including purported jinn possession.

Many Muslims believe that mental illness, just like every struggle in life, can be attributed to Allah (God), either as a result of transgressions, deviation from religious practices, quotidian trials and tribulations, or as a means of purification [6]. Islamic teachings emphasize the interconnectedness of the mind, body, and soul—an unhealthy nafs (soul) can be reflected both in one’s physical body and in one’s psychological state. In Islam, not all mental health issues have a supernatural cause [6]. For example, during medieval times, Europeans associated mental illness with demons, whereas Muslim scholars, including Ibn Sina (Avicenna in the West), a prominent physician regarded as the father of early modern medicine, viewed mental health conditions as physiologically rooted [22, 23].

Many Muslims also believe that diseases serve to purify the body [24]. Consequently, illness is not perceived as solely negative but also as an opportunity for spiritual rewards through resilience and endurance. Comprehending this perspective is paramount for providing effective psychiatric diagnosis and treatment to Muslim patients.

It is believed that ruqyah, through the recitation of the Qur’an, can address a wide spectrum of physical, mental, and spiritual health issues [25]. One purpose of ruqyah is in treating the evil eye, jinn possession, envy, and black magic [15]. Ways, among others, that a jinn can be expelled from the body include remembering God and reciting the Qur’an [26], seeking refuge with God by calling upon him to help the possessed [27], gently blowing into one’s hand while reading the Qur’an and placing the hand on the afflicted’s body [28], and physically blowing into the mouth of the possessed after reading the Qur’an and commanding the jinn to leave.

Reciting the Qur’an during ruqyah can be beneficial in two ways: the spoken words of the Qur’an can exert a powerful healing and protective effect on the listener, because they are considered divine words [25]. The individual reciting the Qur’an can use it to seek guidance and assistance from God, either for their own health or for the well‐being of others [9]. As noted, ruqyah is employed to heal individuals dealing with possession; the words of the Qur’an are used to “expel” the jinn residing within the human body [25].

Building confidence in the patient’s ability to control bad jinn (shaytaan) is crucial in overcoming the fear of jinn and the feeling of being possessed. Muslims often fear jinn because they are unseen and therefore unpredictable. As noted earlier, Muslim scholars distinguish between good jinn and shaytaan [6, p 113], and most believe that jinn are comparable to children in strength and mental ability. Although jinn are believed to be capable of moving heavy objects, adult humans tend to be stronger and possess superior logical reasoning and mental ability. Further, when Muslims recite the Qur’an or seek God’s protection from shaytaan, these jinn are known to flee and hide. Therefore, a strong, educated believer should not fear shaytaan; rather, these jinn should fear the believer.

Khalifa and Hardie [18] indicate that *jinn* possession would appear as “a syndrome consisting of clouding of consciousness, changed demeanor and tone of voice, and subsequent amnesia…true jinn possession can cause a person to have seizures and to speak in an incomprehensible language. The possessed is unable to think or speak from his own will.” These characteristics were not present in our patient, which led the imam to conclude that, contrary to our patient’s belief, he was not possessed by a jinn. For example, the imam pointed out that our patient’s voice did not change in tone or inflection when he spoke as the jinn and said he was 700 years old. Nevertheless, as part of the ruqyah healing ritual, the imam read verses from the Qur’an to our patient, which gave him comfort: “The *jinn* lets me pray now.” Our patient was not uncomfortable with the Qur’an recitation; on the contrary, he enjoyed and was relieved by it—the opposite of how a bad jinn is expected to react.

As well, when the imam told our patient he was not possessed, the patient indicated that what he was experiencing was similar to what he had seen on YouTube videos. The manner in which he made that statement further led the imam to believe that our patient was imitating or trying to experience what he saw on the videos, rather than going through an actual possession experience.

Among 70,000 patients (10,000 Muslims) receiving psychological therapy, those who identified as Muslim were, on average, less likely to achieve reliable recovery from anxiety and depression following “talking therapy” compared to individuals of other, or no, religious identities [29]. These findings do not suggest that psychotherapy lacks benefit for Muslim patients; rather, they highlight the overall importance of incorporating culturally responsive and therapeutic elements aligned with patients’ diverse cultural and religious backgrounds.

It thus is important for mental health treatment teams to grasp the Islamic viewpoint regarding spiritual and mental well‐being in order to consider the role of jinn in Muslim patients’ lives [30–32]. For example, while interviewing our patient, prior to getting the imam involved, the psychiatric team would ask, “Is the *jinn* still inside you?” and “What is the *jinn* saying?” At the time, these questions seemed benign, because they were replacing common questions such as, “Are the voices still there?” and “What are the voices saying?” In retrospect, however, they likely perpetuated the patient’s delusion that he was being possessed. Indeed, the imam advised the psychiatric team to stop using the word, jinn, when speaking with the patient, to help him relinquish his belief of being possessed and focus on his true medical condition.

Our case report highlights some challenges in dealing effectively with patients from different cultures who hold “overvalued ideas.” Differentiating a culturally appropriate religious belief from a psychotic delusion can be difficult, although certain criteria can be helpful, such as the conviction, preoccupation, and impact on functioning of the individual with the belief, as well as its cultural influence [1]. Finding a religious/spiritual leader with the requisite expertise to address the clinical issue and the willingness to do so can be a challenge. And acquiescence of the patient and family to involvement of the religious leader in the patient’s care is required. Whether the religious leader is permitted entrance to the facility, in the case of an inpatient, and whether the religious leader is geographically close to the patient are additional considerations. Fortunately, these challenges were met in the care of our patient.

As pointed out earlier, the growth of the Muslim population globally has increased the need for culturally competent mental health care [6, p 4]. Life stressors include Islamophobia and microaggression, lack of family support, intergenerational tensions around core religious and ethnic values, and general prejudice and racism [6, p 4]. Some Muslims may be reluctant to seek professional mental health help, especially with the cultural stigma surrounding mental illness. Because of fear of cultural insensitivity in mainstream clinicians, Muslims may prefer to consult a faith healer or an imam rather than a mental health professional. Seeking out alternative healing practices secondary to mistrust of healthcare providers, language barriers, differences in culture, and apprehensions surrounding mental health treatment can contribute to decreased utilization of mental health resources in the Muslim community [15] and, when treated, reduced adherence to medication regimens [33]. This is not to suggest that mental health professionals and usual therapeutic modalities employed in the general population should be disregarded: In psychiatry and broader mental health practice, there is a major opportunity to enhance awareness and understanding of cultural differences in Muslim patients so that therapy and treatment can be tailored to their specific belief systems, often in collaboration with spiritual leaders [34].

Other interventions suggested by scholars to provide culturally sensitive treatment to Muslims as well as other patients include holding a nonjudgmental attitude, avoiding condemnation of cultural and spiritual beliefs, and involving the family in patient care; that is, incorporating elements of both cultural competence and cultural humility [35, 36]. There are significant opportunities in psychiatry to increase liaison and referrals between religious leaders and mental health services when caring for Muslim patients. Because religiosity can have both positive and negative effects on treatment adherence in individuals with schizophrenia, it is important to assess religiosity and spirituality in such individuals to understand their “needs and struggles” [37]. Closer cooperation between Muslim spiritual leaders and mental health professionals should provide greater access to psychosocial therapies that are culturally sensitive to Islamic beliefs [15]. Contacting local mosques can be a good first step in locating a knowledgeable imam.

## 4. Clinical Recommendations for Jinn‐Related Delusions

Based on our case and the published literature, the following considerations should help clinicians work effectively with Muslim patients presenting with jinn‐related delusions.

1. Elicit beliefs respectfully: Ask open‐ended, nonjudgmental questions about jinn and spiritual experiences to understand the patient’s perspective, without reinforcing delusions.
2. Assess cultural norms and context: Explore what beliefs are normative within the patient’s family, community, and cultural background and when these beliefs deviate from shared norms. Collateral from family or a trusted cultural broker can help distinguish culturally accepted practices from psychotic symptoms.
3. Collaborate with religious leaders: Engage local imams or other spiritual leaders to be part of the treatment team only with the patient’s informed consent. Sources for locating knowledgeable leaders include mosques, community organizations, and hospital chaplaincy services. Religious leaders should demonstrate cultural humility and an understanding of mental illness, and clear professional boundaries must be maintained.
4. Integrate spiritual consultation with standard psychiatric care: Religious involvement should complement, not replace, evidence‐based pharmacologic and psychotherapeutic interventions.
5. Respond to symptom attribution: When patients attribute experiences to jinn, acknowledge their beliefs without validating delusions, and redirect toward symptom management and safety planning.

## 5. Conclusion

Integration of spirituality into medical treatment can enhance patient outcomes in many specialties [38]. Continuing to integrate spirituality broadly into mental health care will enrich our understanding of human nature and the patient experience and result in better outcomes through holistic medical practice [39]. Understanding the cultural beliefs of psychiatric patients is critical for mental health professionals, allowing them to provide respectful and effective care that fosters trust and results in better health outcomes. Our case highlights the importance of considering the inclusion of a qualified spiritual leader when dealing with religious delusions. In this instance, an imam who was knowledgeable about jinn telling the patient he does not have a jinn inside him carried considerable weight with the patient. Traditionally, ruqyah is best done in person. If, however, qualified imams are not available locally, or if psychiatric units have limitations on who can enter, a virtual interview can be effective, as it was with our patient. By respecting spiritual beliefs and combining them with medical and psychotherapeutic interventions, mental health professionals can create more effective, culturally appropriate care for all patients.

## Author Contributions

All authors contributed to the conception and preparation of this report.

## Acknowledgments

Artificial intelligence was not used in its writing.

## Funding

This report did not receive any funding for its preparation.

## Ethics Statement

The Ventura County Medical Center Institutional Review Board does not require submission of case reports for approval. The case presented here was de‐identified as much as possible, and all HIPAA and other confidentiality requirements have been adhered to.

## Consent

Consent to publish this case report was obtained from the patient.

## Conflicts of Interest

The authors declare no conflicts of interest.

## Data Availability

Data sharing is not applicable—case report details are not publicly available, owing to privacy and ethical restrictions.
